# Simultaneous blockade of IL-6 and CCL5 signaling for synergistic inhibition of triple-negative breast cancer growth and metastasis

**DOI:** 10.1186/s13058-018-0981-3

**Published:** 2018-06-14

**Authors:** Kideok Jin, Niranjan B. Pandey, Aleksander S. Popel

**Affiliations:** 1 Department of Pharmaceutical Sciences, Albany College of Pharmacy and Health Sciences, Albany, NY 12208 USA; 2 Department of Biomedical Engineering, Johns Hopkins University School of Medicine, Baltimore, MD 21205 USA; 3 Department of Oncology and Sidney Kimmel Comprehensive Cancer Center, Johns Hopkins University School of Medicine, Baltimore, MD USA

**Keywords:** Triple negative breast cancer, Tumor microenvironment, Secretome, Drug repurposing, Maraviroc, Tocilizumab

## Abstract

**Background:**

Metastatic triple-negative breast cancer (TNBC) is a heterogeneous and incurable disease. Numerous studies have been conducted to seek molecular targets to treat TNBC effectively, but chemotherapy is still the main choice for patients with TNBC. We have previously presented evidence of the important roles of interleukin-6 (IL-6) and chemokine (C-C motif) ligand 5 (CCL5) in TNBC tumor growth and metastasis. These experiments highlighted the importance of the crosstalk between cancer cells and stromal lymphatic endothelial cells (LECs) in tumor growth and metastasis.

**Methods:**

We examined the viability and migration of MDA-MB-231-LN, SUM149, and SUM159 cells co-cultured with LECs when treated with maraviroc (CCR5 inhibitor) and tocilizumab (anti-IL-6 receptor antibody). To assess the anti-tumor effects of the combination of these two drugs in an athymic nude mouse model, MDA-MB-231-LN cells were implanted in the mammary fat pad and maraviroc (8 mg/kg, orally daily) and cMR16-1 (murine surrogate of the anti-IL-6R antibody, 10 mg/kg, IP, 3 days a week) were administrated for 5 weeks and effects on tumor growth and thoracic metastasis were measured.

**Results:**

In this study, we used maraviroc and tocilizumab to confirm that IL-6 and CCL5 signaling are key pathways promoting TNBC cell proliferation and migration. Further, in a xenograft mouse model, we showed that tumor growth was dramatically inhibited by cMR16-1, the mouse version of the anti-IL6R antibody. The combination of maraviroc and cMR16-1 caused significant reduction of TNBC tumor growth compared to the single agents. Significantly, the combination of maraviroc and cMR16-1 abrogated thoracic metastasis.

**Conclusion:**

Taken together, these findings show that IL-6 and CCL5 signaling, which promote crosstalk between TNBC and lymphatic vessels, are key enhancers of TNBC tumor growth and metastasis. Furthermore, these results demonstrate that a drug combination inhibiting these pathways may be a promising therapy for TNBC patients.

**Electronic supplementary material:**

The online version of this article (10.1186/s13058-018-0981-3) contains supplementary material, which is available to authorized users.

## Background

Lymphangiogenesis plays a critical role in tumor invasion, distal metastasis, and immune unresponsiveness. Crosstalk between tumor-associated lymphatic endothelial cells (LEC) and cancer cells enhances the recruitment of cancer cells to the lymphatic system from primary tumors. This recruitment is increased by various secreted factors such as CCL21, CXCL12, CCL27, IL6 and KAI1 [1], which also induce lymphangiogenesis in tumor-draining lymph nodes (LNs). Lymphangiogenesis, which promotes tumor metastasis, is induced in the pre-metastatic niche by lymphangiogenic growth factors such as vascular endothelial growth factor (VEGF)-C, VEGF-D, angiopoietins, platelet-derived growth factor (PDGF)-BB/AA and basic fibroblast growth factor (bFGF) [2–6]. The recruitment of immune cells such as myeloid-derived suppressor cells (MDSCs), tumor-associated macrophages (TAMs), and immature dendritic cells (DCs) contribute to tumor-induced immunosuppression in the lymph nodes [7]. Due to a paucity of lymphatic markers the role of lymphangiogenesis in tumor growth and metastasis has not been studied as well as the role of blood endothelial cells (BEC). We have previously demonstrated that the crosstalk between LEC secreting CCL5 and triple-negative breast cancer (TNBC) cells expressing CCR5, the CCL5 receptor, promotes the recruitment of TNBC cells towards the lymphatic vessels, induces lymphangiogenesis, and facilitates subsequent lung metastasis. Consistent with these finding, we showed that maraviroc, a CCR5 inhibitor with anti-retroviral activity, inhibited TNBC lymphangiogenesis and lung metastasis. Furthermore, we discovered that IL-6, which is secreted by TNBC cells, is a key factor in upregulating CCL5 expression in LECs by activating the IL6 receptor and subsequently STAT3, which enhances transcription of the *CCL5* gene. In our earlier study, we showed that inhibiting the IL-6 signaling pathway by depleting IL-6 levels using an anti-IL-6 antibody or a STAT3 inhibitor decreased CCL5 expression and consequently lymphogenous metastasis [8]. Here, we present more evidence that both IL-6 and CCL5 are key factors in TNBC lymphangiogenesis, tumor growth, and thoracic metastasis. Furthermore, by using maraviroc (CCR5 inhibitor) and cMR16-1 Ab (murine surrogate of the anti-IL-6 receptor antibody) we showed that simultaneous blockade of CCR5 and IL-6 receptor signaling strongly inhibits TNBC tumor growth and profoundly inhibits TNBC tumor metastasis.

## Methods

### Cell lines

MDA-MB-231-luc-D3H2LN (MDA-MB-231-LN) cells were purchased from Caliper and propagated in RPMI-1640 medium supplemented with 10% FBS and 1% penicillin/streptomycin (Sigma). SUM149 and SUM159 breast cancer cells were cultured in F-12 medium supplemented with 5% FBS, 1 ng/ml hydrocortisone, 5 μg/ml insulin (Sigma, St. Louis, MO, USA), and 0.1 mM HEPES (ThermoFisher Scientific, Waltham, MA, USA). LECs were purchased from Lonza, and grown in EGM-2MV. Cells were maintained under standard conditions of 37 °C and 5% CO^2^. Cells were cultured for a maximum of 4 weeks after thawing fresh, early passage cells and confirmed to be Mycoplasma negative (Hoechst stain).

### Conditioned medium

When TNBC cells had grown to confluence in T175 tissue culture flasks, the normal cancer cell growth medium was replaced with 8 ml serum-free medium (SFM) after extensive washing. After 24 h incubation in a tissue culture incubator, the supernatant was centrifuged and filtered through 0.2-μm syringe filters (Corning). The resulting tumor-conditioned medium (TCM) was stored in aliquots at − 80 °C. When LECs reached 30–40% confluence in T75 tissue culture flasks, the growth medium (GM) was replaced with 30% TCM in GM (TCM:GM = 3:7) to allow the TCM to activate the LECs. For education of LECs by TCM, the cells were allowed to grow in the medium for 3–4 days at which point the medium was replaced with 3 ml SFM containing 2% FBS. After 48 h, the supernatant was centrifuged and filtered. The resulting tumor-educated LEC conditioned medium, (TCM-LEC)CM, was stored in aliquots at − 80 °C to avoid multiple freeze thaws.

### Cell migration and proliferation assays

Cancer cell migration was assessed using the Oris™ cell migration kit (Platypus), as previously described [8]. The (TCM-LEC)CM (100 μl) with or without 20 μM maraviroc (R&D Systems) or 200 μg/ml tocilizumab (Genentech) was added once the cancer cells had attached. Migration and proliferation assays using CIM (cell invasion and migration) plates and the RTCA system (ACEA Bioscience) were performed as previously described [9].

### Mouse xenograft studies

Before tumor inoculation, athymic nude mice (female, 5–6 weeks, 18–20 g) were pre-treated by injecting 50 μl TCM or SFM subcutaneously for 2 weeks daily as described previously [8]. MDA-MB-231-LN cells were grown to 90% confluence, trypsinized, resuspended in serum-free medium, and mixed 1:1 with Matrigel (BD Biosciences) and 2 × 10^6^ cells were injected into the upper inguinal mammary fat pad of the animals induced for 2 weeks with TCM as described above. Tumor sizes were measured using calipers, and the volume was calculated, using the formula: V = 0.52 × (length) × (width)^2^. Animals were imaged every week to track anterior tumor metastases, using the IVIS Xenogen 200 optical imager (Xenogen) after intraperitoneal (i.p.) injection of D-luciferin (Caliper, 150 mg/kg body weight). After 5 weeks, organs were harvested and bathed in D-luciferin solution for 5–10 min and placed in the IVIS imager to detect metastases ex vivo. Luciferase-mediated photon flux was quantified using Living Image® 3D Analysis (Xenogen). Maraviroc (8 mg/kg body weight, R&D systems) was administered orally daily; cMR16-1 (10 mg/kg, Genentech) was administered intraperitoneally 3 days per week for 5 weeks.

### Immunofluorescence and immunohistochemical analysis

Immunofluorescence and immunohistochemical analysis (IHC) were performed using monoclonal antibodies against CD31 and pan-cytokeratin (Sigma). For immunofluorescence, after blocking with 5% normal goat or normal chicken serum (Jackson Immunoresearch) in PBS/Tween (PBST) (0.3% Triton) for 1 h at room temperature (RT), the sections were treated with anti-CD31 primary antibodies overnight at 4 °C. After three rinses with PBST, the sections were incubated for 1 h at RT with FITC-conjugated goat anti-rabbit secondary antibodies (1:500). After three rinses with PBST, the samples were counterstained with 4’ ,6-diamidino-2-phenylindole (DAPI) (1:10,000, Roche) (5 min at RT). The samples were washed with PBST once and mounted with the ProLong Gold anti-fade reagent (Invitrogen) in the dark. Fluorescent signals were visualized and digital images were obtained using the Zeiss LSM-700 confocal microscope (Carl Zeiss). For IHC, after blocking with 5% goat serum in PBST for 1 h at room temperature, the sections were treated with the pan-cytokeratin antibody overnight at 4 °C, then the peroxidase conjugated streptavidin complex method was performed, followed by the 3, 3′ diaminobenzidine (DAB) procedure according to the manufacturers’ protocols (VECTASTAIN Elite ABC Kit, Vector Lab).

### Statistical analysis

Error bars represent the SEM. All statistical tests were two sided, and differences were considered statistically significant at *P* < 0.05. The synergy calculation was performed using the Chou-Talalay method as previously described [10–12].

## Results

### Maraviroc and tocilizumab block TNBC cell proliferation

**Fig. 1:**
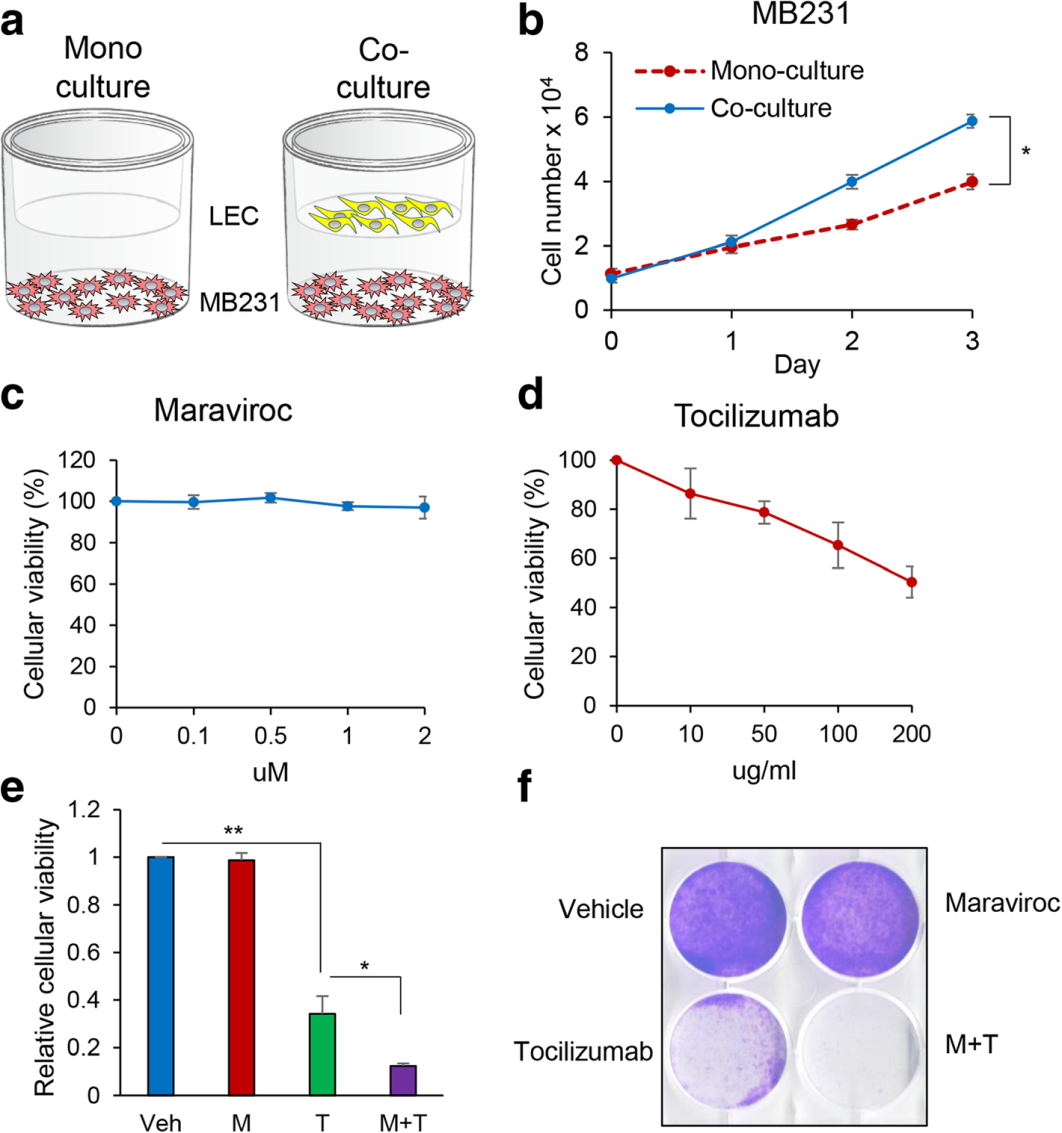
Co-culture of MDA-MB-231-LN cells with lymphatic endothelial cells (LECs) promotes cell proliferation, which is inhibited by tocilizumab. **a** Schematic diagram of co-cultured MDA-MB-231-LN cells with LECs (RTCA system, ACEA Biosciences Inc.). **b** Proliferation assays of co-cultured MDA-MB-231-LN cells on the bottom chambers with LECs (10,000 cells per well) on the top chamber were performed for 3 days. Cells were trypsinized and manually counted with a hemocytometer. Results are means ± SEM (*n* = 3). **c** Cellular viability assays of co-cultured MDA-MB-231-LN cells on the bottom chambers with LECs on the top chamber in treatment with various concentration of maraviroc and **d** tocilizumab (E-plates). The bottom and top chambers were combined, loaded in the RTCA system and the cell index was measured continuously for 48 h (*P < 0.001, *n* = 3). **e** The same assay was performed as in **d** in treatment with maraviroc (2 uM) (M), tocilizumab (200 μg/ml; 135 uM) (T), and the combination of maraviroc and tocilizumab (M+T); Veh, Vehicle. **f** Crystal violet staining assay was performed as in **e**

We have previously shown that lymphatic endothelial cells (LECs) promote TNBC tumor growth [13]. Furthermore, we have discovered that IL-6 secreted by TNBC cells binds to the IL-6 receptor on LECs and activates the STAT3 signaling pathway. The phosphorylated ternary complex of STAT3, c-Jun, and ATF-2 binds to a CRE site on the CCL5 promoter to enhance CCL5 transcriptional activity in LECs. The CCL5 secreted by LECs recruits CCR5-positive TNBC cells and guides them towards the lymphatic system [8]. This finding motivated the hypothesis that inhibition of both CCL5 and IL-6 signaling would attenuate TNBC tumor growth and thoracic metastasis. In our earlier work we elucidated the role of IL-6 in TNBC growth and metastasis by depleting its levels in the TCM administered to the animals prior to tumor inoculation. To facilitate translation of our work to patients, here we tested the effect of an antibody against the IL-6 receptor on the growth and metastasis of TNBC tumors. We examined the viability of MDA-MB-231-LN cells co-cultured with LECs in the presence of maraviroc (CCR5 inhibitor, Pfizer) and tocilizumab (anti-IL-6 receptor antibody, Genentech) (Fig. 1a). The proliferation of MDA-MB-231-LN cells was increased when co-cultured with LECs (Fig. 1b). We observed that maraviroc had no significant effect, whereas tocilizumab decreased MDA-MB-231-LN cellular viability in a concentration-dependent manner (Fig. 1c and d). Using transwell plates, real-time co-culture E-plates (RTCA system ACEA Biosciences Inc.) and crystal violet staining, we confirmed that tocilizumab inhibited the cell viability of three different TNBC cells including MDA-MB-231-LN, SUM149, and SUM159 compared to control and maraviroc. The viability of TNBC cells was decreased further in the combination of maraviroc and tocilizumab compared to the single agents as seen above. Furthermore, we analyzed tocilizumab with maraviroc in the TNBC cellular viability according to the Chou-Talalay method for drug synergy analysis. We found that the combination of maraviroc and tocilizumab is highly synergistic with CI < 1 (Fig. 1e, f, and Additional file 1: Figure S1). These results suggest that the crosstalk between TNBC cells and LECs enhances TNBC cell proliferation, and that the IL-6 signaling plays a critical role in TNBC cellular viability.

### Decrease of TNBC cell migration by inhibition of IL-6 and CCL5 signaling

**Fig. 2:**
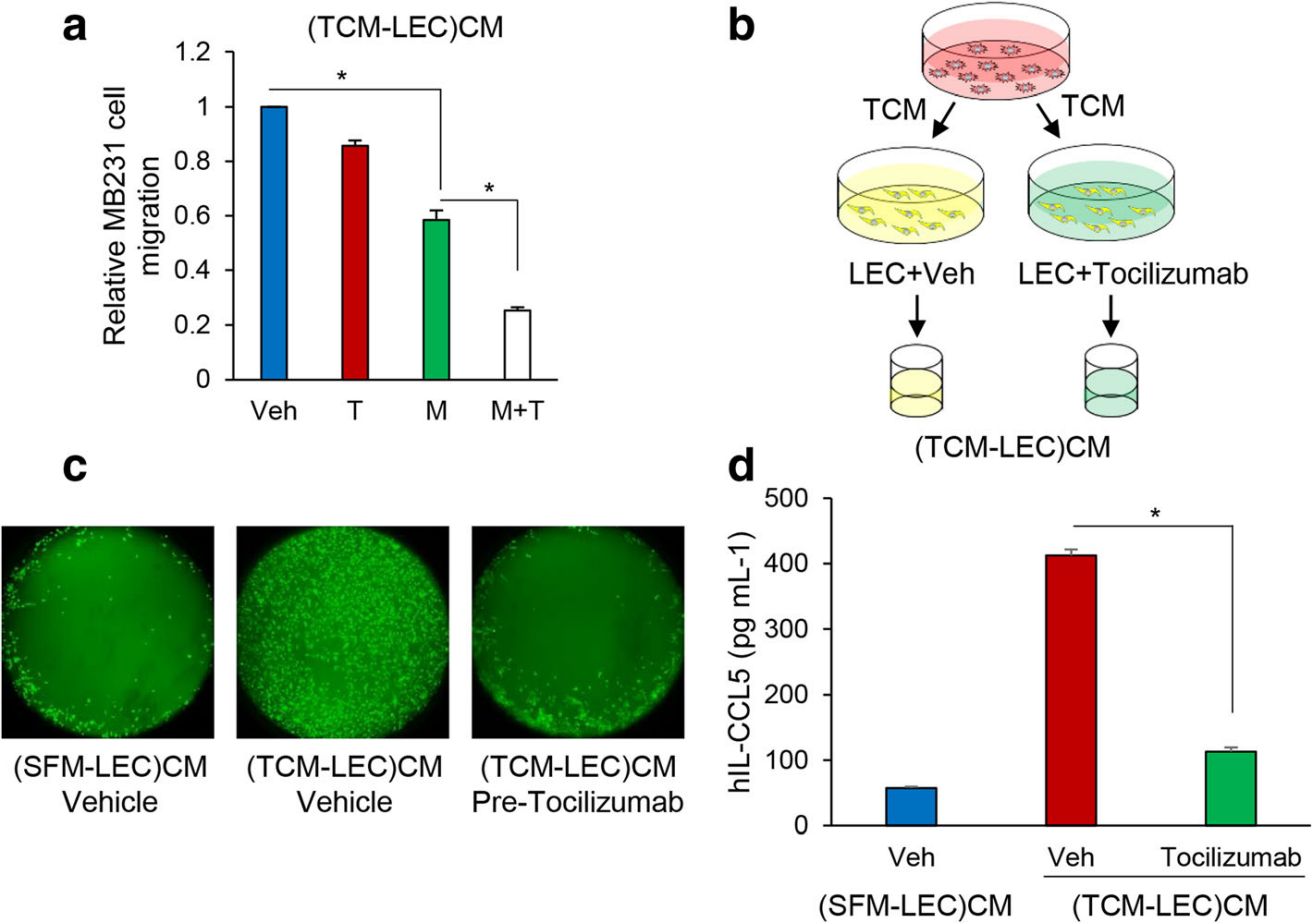
The combination of maraviroc and tocilizumab inhibits MDA-MB-231-LN cell migration. **a** Migration assay of MDA-MB-231-LN cells (top chamber) in treatment with maraviroc (M), tocilizumab (T), and the combination of maraviroc and tocilizumab (M+T) with 180 ul of conditioned medium (CM) (bottom chamber) from lymphatic endothelial cells (LECs) cultured with serum-free medium (SFM) or tumor-conditioned medium (TCM) of MDA-MB-231-LN cells in the RTCA system. The cell index was measured continuously for 48 h. The representative migration is shown (*P < 0.001, *n* = 3). **b** Schematic diagram of (TCM-LEC)CM. CM was prepared by growing LECs in 30% SFM or TCM of MDA-MB-231-LN cells with treatment with either vehicle or tocilizumab for 4 days and the medium was replaced with 3 ml SFM with 2% FBS. After 48 h, the supernatant was centrifuged and filtered. The conditioned medium, (TCM-LEC)CM was used for the migration assay. **c** Migration assay of MDA-MB-231-LN cells pre-labeled with Cell Tracker Green and the migration was measured using the Oris cell migration kit. Labeled MDA-MB-231-LN cells (50,000) in complete medium were added to each well of a 96-well plate containing stoppers to prevent the cells from settling in the center region of the wells. Cells were allowed to adhere for 24 h, after which the stoppers were carefully removed. CM as described in **b** was added, and the cells that migrated to the center of the well were observed for 48 h. **d** ELISA of human CCL5 (Quantikine ELISA, R&D System) in the CM described in **b** (*P < 0.001, *n* = 3). Veh, Vehicle

In previous studies, we showed that the TCM from TNBC cells induced LECs, and the conditioned medium from LECs induced by TCM, (TCM-LEC)CM, facilitated TNBC cell migration. We found that IL-6 in TCM plays an important role in upregulating CCL5 expression in LECs. The CCL5 in (TCM-LEC)CM plays a key role in TNBC cell motility and maraviroc efficiently inhibited this migratory effect [8]. Here we investigated the effect of the combination of maraviroc and tocilizumab on TNBC cell migration in conditioned medium from LECs induced by TCM of TNBC cells, (TCM-LEC)CM, using a CIM-plate from ACEA Biosciences. We cultured TNBC cells (top chamber) with (TCM-LEC)CM (bottom chamber) in treatment with maraviroc, tocilizumab, and the combination of maraviroc and tocilizumab, and measured the migration of TNBC cells in the RTCA system. Maraviroc significantly inhibited TNBC cell migration by inhibiting CCR5 of TNBC cells in response to (TCM-LEC)CM including CCL5 compared to control. Compared to maraviroc, tocilizumab had less effect on the migration of TNBC cells by blocking IL-6 receptor in TNBC cells. Significantly, TNBC cell migration was decreased by the combination of maraviroc and tocilizumab, about three fold compared to treatment with maraviroc alone. It implied that both CCR5 and IL-6 receptor signaling in TNBC cells play an important role in TNBC cell migration (Fig. 2a and Additional file 1: Figure S2A and B). We wanted to confirm that TNBC cell migration depended on the crosstalk between TNBC cells and LECs through IL-6 and CCL5 signaling so LECs were cultured in TCM of TNBC cells pre-treated with tocilizumab (scheme shown in Fig. 2b). Inhibition of the IL-6 signaling pathway by tocilizumab should result in reduced levels of CCL5. We found that TNBC cell migration was enhanced in (TCM-LEC)CM compared to (SFM-LEC)CM as expected. However, we found that TNBC cell migration was significantly inhibited in tocilizumab-pre-treated (TCM-LEC)CM compared to control (TCM-LEC)CM (Fig. 2c and Additional file 1: Figure S2C). As expected, the secretion of CCL5 was decreased in tocilizumab-pre-treated (TCM-LEC)CM compared to control (TCM-LEC)CM (Fig. 2d and Additional file 1: Figure S2D and E). These results show that crosstalk between TNBC cells and LECs mediated by IL-6 and CCL5 signaling is important for TNBC cell migration.

### The combination of maraviroc and cMR16-1, an anti-mouse anti-IL6 receptor antibody, inhibits growth of MDA-MB-231-LN tumors

**Fig. 3:**
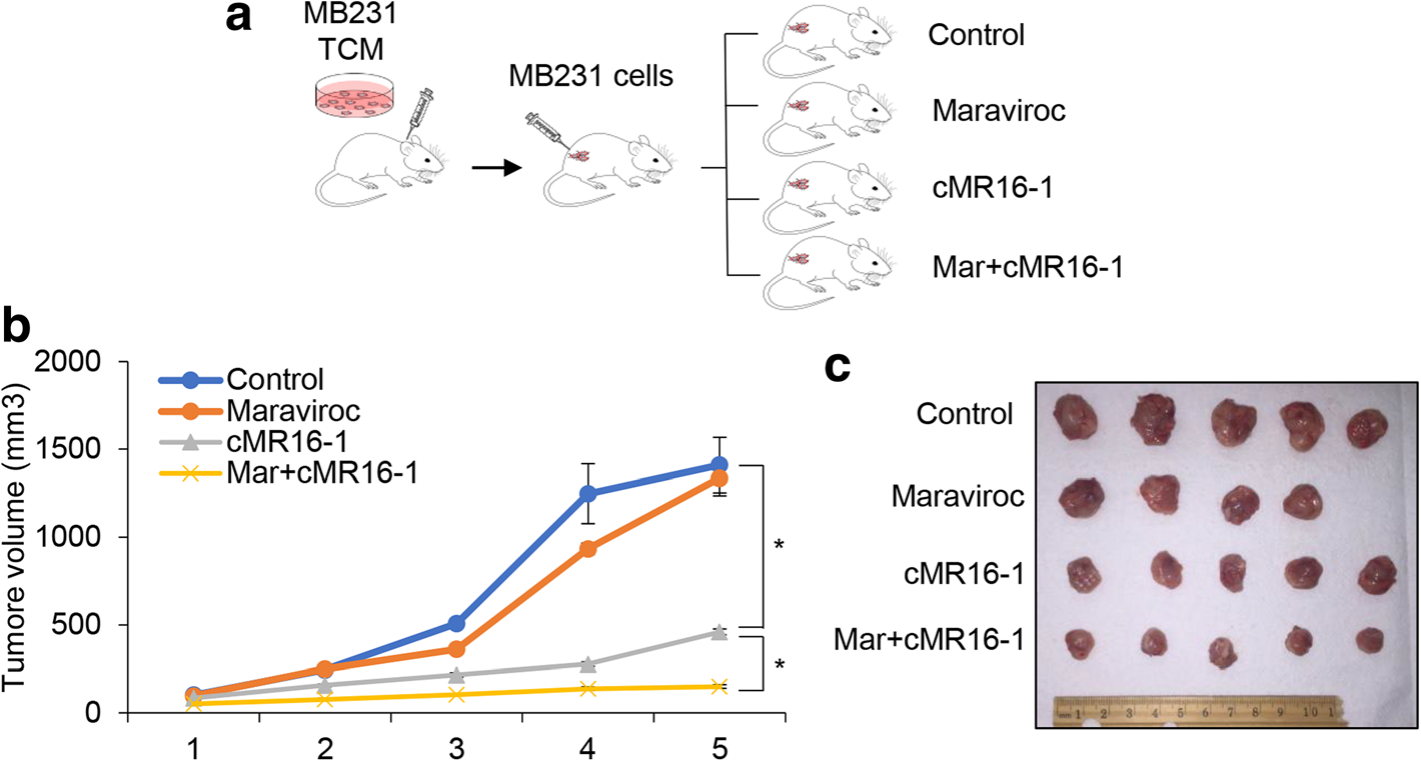
MDA-MB-231-LN cell tumor growth was inhibited by the combination of maraviroc and tocilizumab. **a** Generation of MDA-MB-231-LN cell xenografts administered maraviroc, tocilizumab, and the combination of maraviroc and tocilizumab. **b** Tumor growth curves of MDA-MB-231-LN cells implanted mammary fat pad in athymic mice treated with maraviroc (8 mg/kg body weight, R&D systems) orally daily; cMR16-1 (10 mg/kg, Genentech) intraperitoneally 3 days per week for 5 weeks (mean ± SEM, **P < 0.005, *n* = 10). **c** Size of MDA-MB-231-LN cells xenografts in athymic nude mice after 5 weeks of treatment with maraviroc and cMR16-1. TCM, tumor-conditioned medium

Next we investigated the inhibitory effect of maraviroc and tocilizumab on the growth of orthotopic MDA-MB-231-LN tumors in athymic mice. We administered maraviroc (8 mg/kg body weight, orally daily) and cMR16-1 (murine surrogate of the anti-IL-6R antibody, 10 mg/kg body weight, i.p., 3 days a week), and monitored tumor growth for 5 weeks (Fig. 3a). We used the cMR16-1 in these experiments because the LEC cells on lymphatic vessels, which are from the mouse host, carry the mouse IL6 receptor. The growth of MDA-MB-231-LN tumors treated with cMR16-1 was significantly inhibited compared to control while maraviroc had no significant effect on tumor growth. The combination of maraviroc and cMR16-1 enhanced the inhibition of MDA-MB-231-LN cell tumor growth significantly (Fig. 3b and c) compared to cMR16-1 alone. These results demonstrate that the IL-6 receptor is an attractive target in TNBC and that the combination of maraviroc and an anti-IL6 receptor antibody could be beneficial in the treatment of TNBC.

### Inhibition of IL-6 and CCL5 signaling prevents TNBC metastasis

**Fig. 4:**
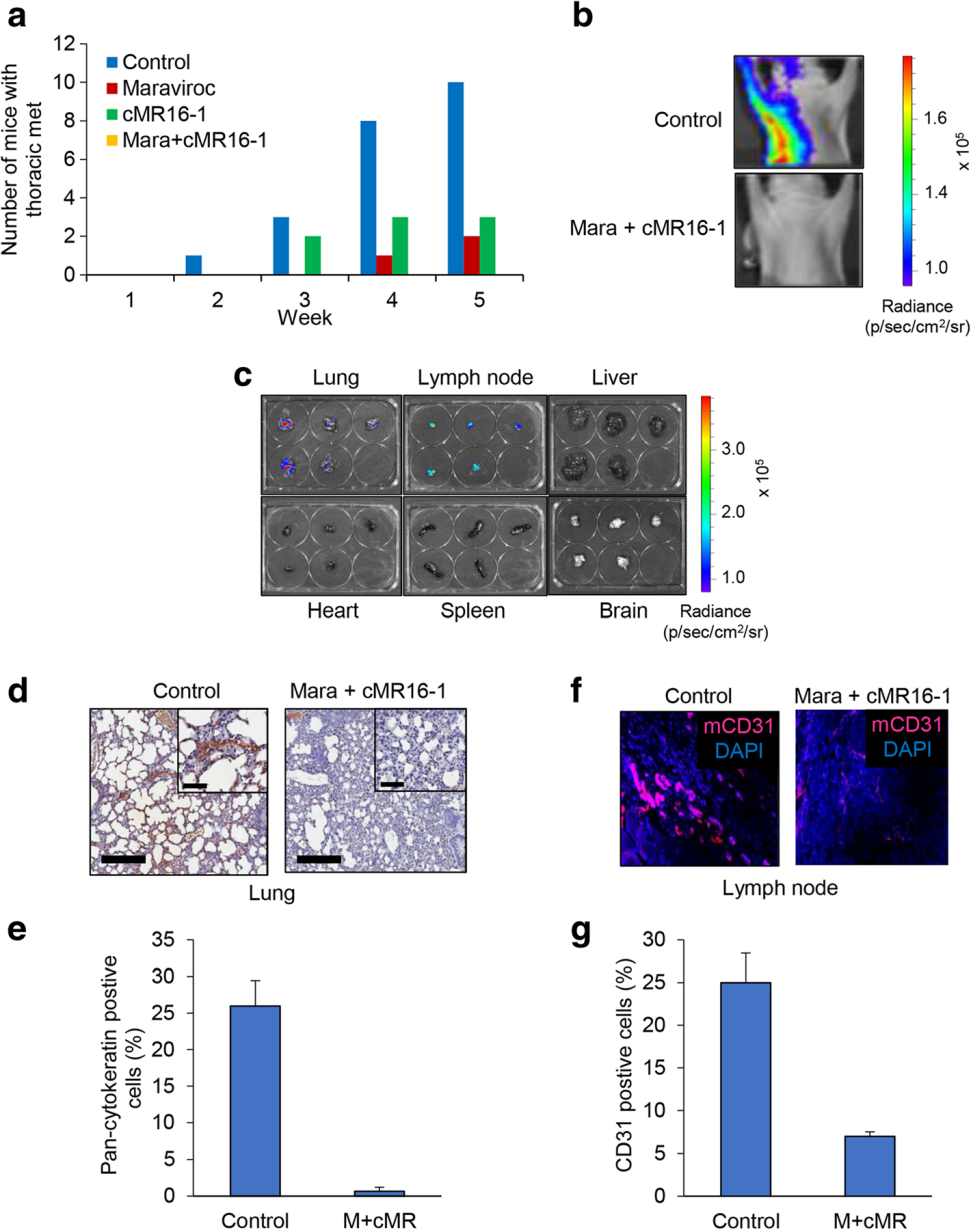
The thoracic metastasis of MDA-MB-231-LN cell tumor was inhibited by the combination of maraviroc and tocilizumab. **a** Athymic nude mice (4–5 weeks, female, from Charles river) were pretreated with tumor-conditioned medium (50 ul) of MDA-MB-231-LN cells for 2 weeks before inoculation with MDA-MB-231-LN cells. After maraviroc and tocilizumab were administered for 5 weeks, the number of mice with thoracic metastasis was counted using the IVIS imager. The incidence of thoracic metastasis was significantly effective in the combination treatment compared to both single agents (*P* < 0.0325, Fisher’s exact test). **b** Representative thoracic metastasis in the control group and the group treated with a combination of maraviroc and tocilizumab as demonstrated by the IVIS imager. **c** Representative organ images of luciferase-mediated photon flux from lung, lymph node, liver, heart, spleen and brain. **d** Formalin-fixed paraffin embedded tissues from the tumor in the control and the combination of maraviroc (Mara) and cMR16-1 groups were used for immunohistochemistry (IHC) with anti-cytokeratin antibodies on the lungs to indicate metastatic colonies. Low-power microscopes are at × 10 magnification (scale bar 200 um). High-power insets are × 40 magnification (50 um). **e** The IHC results were examined and quantified by ImageJ. **f** Representative immunofluorescence images of mouse CD31 in the lymph node and **g** quantification of results

In previous work, we developed a metastatic mouse model in which TCM of MDA-MB-231-LN cells was administered to mice for 2 weeks subcutaneously prior to tumor inoculation. MDA-MB-231-LN cells were then injected into the mammary fat pad to establish orthotopic tumor xenografts [14]. The primary tumors robustly gave rise to thoracic metastases including to the lymph nodes (LNs) and lungs within 4–5 weeks [8]. Utilizing this mouse model, we monitored the effect of maraviroc and cMR16-1 on thoracic metastasis of MDA-MB-231-LN tumors by weekly IVIS imaging over a period of 5 weeks. We observed that MDA-MB-231-LN cell tumors metastasized to the LNs and thoracic region in all the mice of the control group (10/10) within 5 weeks as shown by the increase in photon flux in these tissues. However, thoracic metastases were found in only 20% of the mice (2/10) in the maraviroc treated group and in 30% of the mice (3/10) in the cMR16-1 treated group. Remarkably, we observed no metastasis in LNs and in thoracic tissues in the group of mice treated with the combination of maraviroc and cMR16-1 (Fig. 4a-c). We assayed for the presence of the lung metastasis by IHC with cytokeratin in the lung (Fig. 4d and e) and the lymphangiogenesis in LNs by immunostaining with CD31 (Fig. 4f and g). The results demonstrate that IL-6 and CCL5 signaling in the crosstalk of TNBC cells with LECs plays a key role in metastasis and drugs targeting them could be used to minimize metastasis in patients with TNBC.

## Discussion

Tumor cells are commonly spread to tumor-draining lymph nodes in many types of cancer. There is ample evidence that lymphangiogenesis is actively induced by tumor cells and lymphangiogenic growth factors play an important role in lymphangiogenesis and distal organ metastasis [15–17]. For example, the proteolytic matured VEGF-C and VEGF-D interact with VEGFR-3 and upregulate lymphangiogenic activity [18, 19]; the VEGF-A-VEGFR-2 axis also induces tumor lymphangiogenesis by inducing LEC proliferation [20, 21]. Angiogenin 1 (Ang1) and Ang2 were also shown to be important for stimulating lymphangiogenesis and lymphatic metastasis in a tumor xenograft model [22, 23]. Hepatocyte growth factor receptor (HGF c-MET) is also known to be an inducer of lymphangiogenesis in vivo [24–26]. Studies by our laboratory and others showed that fibroblast growth factor (FGF), epidermal growth factor (EGF), PDGF, and insulin-like growth factor (IGF) play critical roles in lymphangiogenesis [13, 27–30]. In our previous studies, we have shown that crosstalk between MDA-MB-231-LN cells and LECs induce upregulation of EGF to promote MDA-MB-231-LN cell proliferation and LECs co-injected with MDA-MB-231-LN cells into nude mice enhanced tumor growth. In addition, we demonstrated that an interaction of MDA-MB-231-LN and LEC stimulated PDGF-BB expression and recruited pericytes to the neovasculature in a xenograft mouse model; SU16f, a PDGFRβ inhibitor, inhibited the recruitment of pericytes in this model [13]. It has been reported that the CCL5-CCR5 axis enhanced metastasis of basal breast cancer cells [31–33], but detailed mechanisms of how this happens are not yet clear. We have tried to clarify these mechanisms in this and earlier studies. In a previous study, we discovered that IL-6 secreted by TNBC cells upregulates CCL5 and VEGF in LECs through the IL-6-STAT3 signaling pathway. Additionally, we showed that the secreted CCL5 recruited CCR5-positive TNBC breast cancer to LNs resulting in LN angiogenesis and lung metastasis. In addition, the increased levels of VEGF induced LN angiogenesis and supported tumor cell extravasation into the lung. In agreement with these results, we have also found a significant inhibitory effect on TNBC metastasis by the following manipulations: depleting IL-6 in the tumor-conditioned medium injected into mice prior to tumor inoculation using an anti-IL-6 antibody, inhibiting CCL5 by maraviroc, inhibiting VEGF by an anti-VEGF antibody, and inhibiting STAT3 by S3I-201 [8]. Maraviroc exerts its anti-retroviral activity by inhibiting the CCR5 receptor and it is commonly utilized as a treatment for HIV infection. Our results show that maraviroc inhibits TNBC metastasis so we propose repurposing this drug to treat patients with TNBC. In order to produce even more effective treatments for TNBC using repurposed drugs, we tested tocilizumab, a Food and Drug Administration (FDA)-approved anti-inflammatory IL-6 receptor inhibitor drug for the treatment of rheumatoid arthritis. Application of these two drugs allowed us to directly test the hypothesis that drugs against both IL-6 and CCL5 could be used to treat TNBC. We discovered that co-cultures of MDA-MB-231-LN cells with LECs enhanced proliferation of MDA-MB-231-LN cells. It has been reported that autocrine IL-6 is a key promoter of TNBC cell proliferation [34, 35]. In support of our hypothesis, in our experiments, tocilizumab significantly decreased the viability of TNBC cells and the combination of tocilizumab and maraviroc decreased cellular viability even more (Fig. 1). Consistent with this result, we observed no significant difference in the viability of TNBC cells in the presence of maraviroc. Our data demonstrate that IL-6 signaling in the crosstalk between TNBC cells and LECs is a major determinant of TNBC cell proliferation and viability.

We next investigated the effect of maraviroc and tocilizumab on the migration of TNBC cells induced by (TCM-LEC)CM. The results indicate that the migration of TNBC cells is dependent on CCL5-CCR5 signaling and that IL-6 indirectly contributes to migration of TNBC cells by regulating CCL5 expression in LECs (Fig. 2). We tested agents targeting the IL-6 and CCR5 pathways in athymic mice with TNBC tumor xenografts to extend these findings. The growth of MDA-MB-231-LN tumors was inhibited by cMR16-1, whereas maraviroc had no effect on tumor growth, consistent with our observation that maraviroc had no effect on the proliferation of MDA-MB-231-LN cells. The combination of maraviroc and cMR16-1 resulted in greater inhibition of tumor growth compared to the single agents (Fig. 3). Next, we were interested in determining whether maraviroc and cMR16-1 could inhibit TNBC tumor metastasis. Thoracic metastasis was detected in all control group mice while mice treated with either maraviroc or cMR16-1 had low incidences of metastasis. Strikingly, we observed no metastasis in any of the mice treated with a combination of maraviroc and cMR16-1 (Fig. 4). These observations demonstrate that CCL5-CCR5 and IL-6-IL-6R signaling between TNBC cells and LECs plays a critical role in TNBC tumor growth and metastasis.

**Fig. 5:**
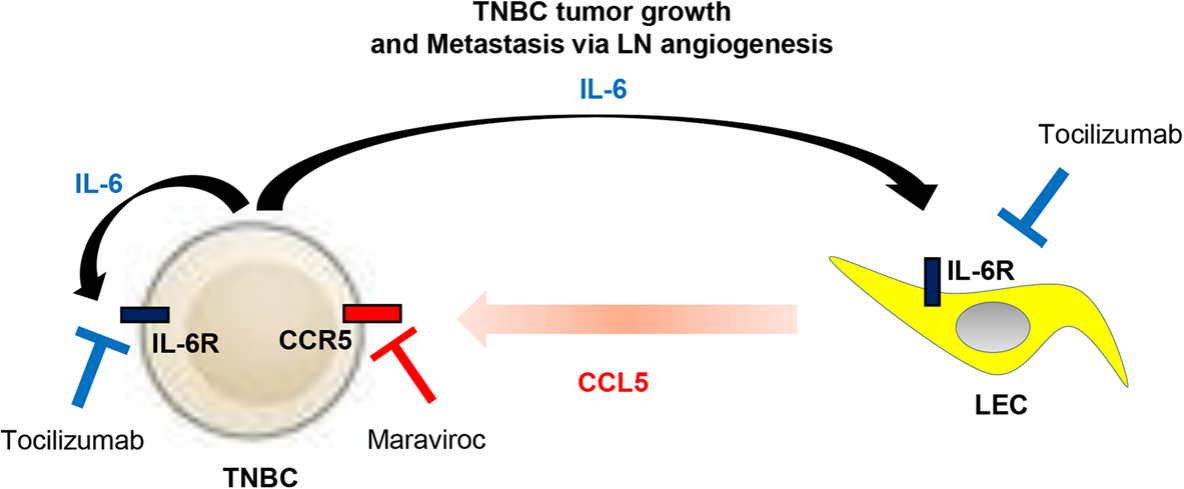
Model of crosstalk between triple negative breast cancer (TNBC) and lymphatic endothelial cells (LECs). In TNBC, secreted IL-6 from TNBC cells binds to IL-6 receptor in LECs, which enhances CCL5 secretion by activating the STAT3 signaling pathway in LECs. The interaction of CCL5 and CCR5 consequently promotes TNBC tumor growth and metastasis. Therefore, maraviroc and tocilizumab are potential therapeutic drugs to inhibit TNBC tumor growth and metastasis

Evidence from our previous studies suggests that TNBC cells secrete IL-6, which interacts with the IL-6 receptor on tumor-associated lymphatic vessels in the tumor microenvironment or on lymphatic vessels in distant organs and that the activated IL-6 receptor stimulates the STAT3 signaling pathway, which results in upregulated CCL5 expression and secretion by LECs. As a result, CCL5 induces LN angiogenesis, and lung and thoracic metastasis. We propose using maraviroc and tocilizumab to block TNBC metastasis through the lymphatic system (Fig. 5).

## Conclusions

Taken together, these studies confirmed that IL-6 and CCL5 function as critical molecules in TNBC growth and metastasis. Furthermore, maraviroc and tocilizumab could be repurposed and provide a new clinical approach to treat TNBC.

## Additional file

Additional file 1: **Figure S1.** Cellular viability assays of co-cultured SUM149 (**A**) and SUM159 (**B**) cells on the bottom chambers with LECs on the top chamber in treatment with various concentration of maraviroc and d tocilizumab (transwell plates). The cellular viability was measured for 72 h by MTT assay (*P < 0.001, *n* = 3). **C** Crystal violet staining assay was performed in treatment with maraviroc (2 uM), tocilizumab (200 μg/ml), and the combination of maraviroc and tocilizumab. **Figure S2.** Migration assay of SUM149 (**A**) and SUM159 (**B**) cells (top chamber) in CM (bottom chamber) from LECs co-cultured with two TNBC cells with treatment with maraviroc, tocilizumab, and the combination of both. The migrated cells were counted for 24 h by the crystal violet staining. The representative migration is shown. (**P < 0.001, *n* = 3). **C** Migration assay of TNBC cells in CM from LECs co-cultured with TNBC cells with tocilizumab pre-treatment. The cells were pre-labeled with Cell Tracker Green and the migration was measured using the Oris cell migration kit. ELISA of human CCL5 (Quantikine ELISA, R&D System) in the CM of LECs co-cultured with SUM149 (**D**) and SUM159 (**E**) cells pre-treated tocilizumab (*P < 0.001, *n* = 3). (PDF 271 kb)

## Abbreviations

Ang: Angiogenin
BEC: Blood endothelial cell
CCL5: Chemokine (C-C motif) ligand 5
CCR5: CCL receptor 5
CIM: Cell invasion and migration
CM: Conditioned medium
c-MET: Hepatocyte growth factor receptor
DCs: Immature dendritic cells
EGF: Epidermal growth factor
ELISA: Enzyme-linked immunosorbent assay
FBS: Fetal bovine serum
FGF: Fibroblast growth factor
GM: Growth medium
HGF: Hepatocyte growth factor
IGF: Insulin-like growth factor
IL-6: Interleukin-6
i.p.: Intraperitoneal/intraperitoneally
LECs: Lymphatic endothelial cells
LNs: Lymph nodes
PBS: Phosphate-buffered saline
PDGF: Platelet-derived growth factor
RT: Room temperature
SFM: Serum-free medium
STAT3: Signal transducer and activator of transcription 3
TAMs: Tumor-associated macrophages
TCM: Tumor-conditioned medium
TNBC: Triple-negative breast cancer
VEGF: Vascular endothelial growth factor
VEGFR: Vascular endothelial growth factor receptor
