# Supplementary material for: Simultaneous blockade of IL-6 and CCL5 signaling for synergistic inhibition of triple-negative breast cancer growth and metastasis
Source: Breast Cancer Res. 2018 Jun 14;20:54. doi: 10.1186/s13058-018-0981-3 (PMC6000947; doi:10.1186/s13058-018-0981-3)
Supplement: Supplementary file 1 — Figure S1. Cellular viability assays of co-cultured SUM149 (A) and SUM159 (B) cells on the bottom chambers with LECs on the top chamber in treatment with various concentration of maraviroc and d tocilizumab (transwell plates). The cellular viability was measured for 72 h by MTT assay (*P < 0.001, n = 3). C Crystal violet staining assay was performed in treatment with maraviroc (2 uM), tocilizumab (200 μg/ml), and the combination of maraviroc and tocilizumab. Figure S2. Migration assay of SUM149 (A) and SUM159 (B) cells (top chamber) in CM (bottom chamber) from LECs co-cultured with two TNBC cells with treatment with maraviroc, tocilizumab, and the combination of both. The migrated cells were counted for 24 h by the crystal violet staining. The representative migration is shown. (**P < 0.001, n = 3). C Migration assay of TNBC cells in CM from LECs co-cultured with TNBC cells with tocilizumab pre-treatment. The cells were pre-labeled with Cell Tracker Green and the migration was measured using the Oris cell migration kit. ELISA of human CCL5 (Quantikine ELISA, R&D System) in the CM of LECs co-cultured with SUM149 (D) and SUM159 (E) cells pre-treated tocilizumab (*P < 0.001, n = 3). (PDF 271 kb) [file 13058_2018_981_MOESM1_ESM.pdf]

Figure S1

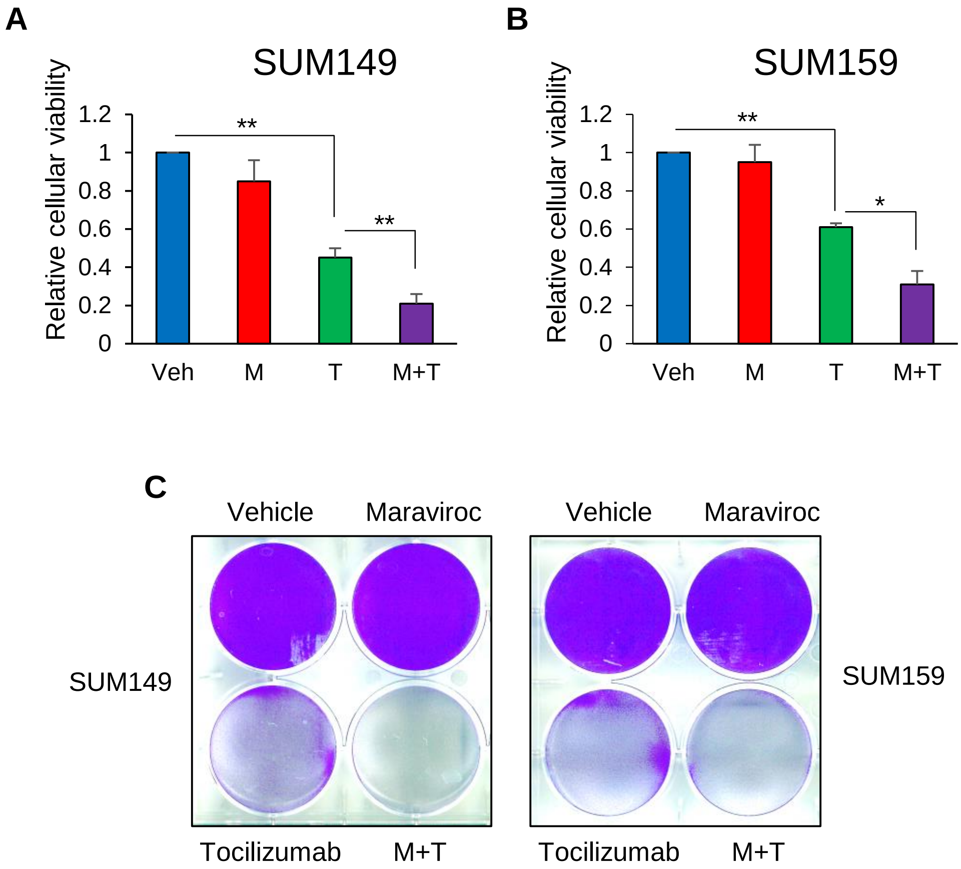

Figure S1: Cellular viability assays of co-cultured **a** SUM149 and **b** SUM159 cells on the bottom chambers with LECs on the top chamber in treatment of various concentration of maraviroc and d tocilizumab (Transwell plates). The cellular viability was measured for 72 h by MTT assay (*P<0.001, n=3). **c** Crystal violet staining assay was performed in treatment of maraviroc (2 uM), tocilizumab (200 ug/ml), and the combination of maraviroc and tocilizumab.

Figure S2

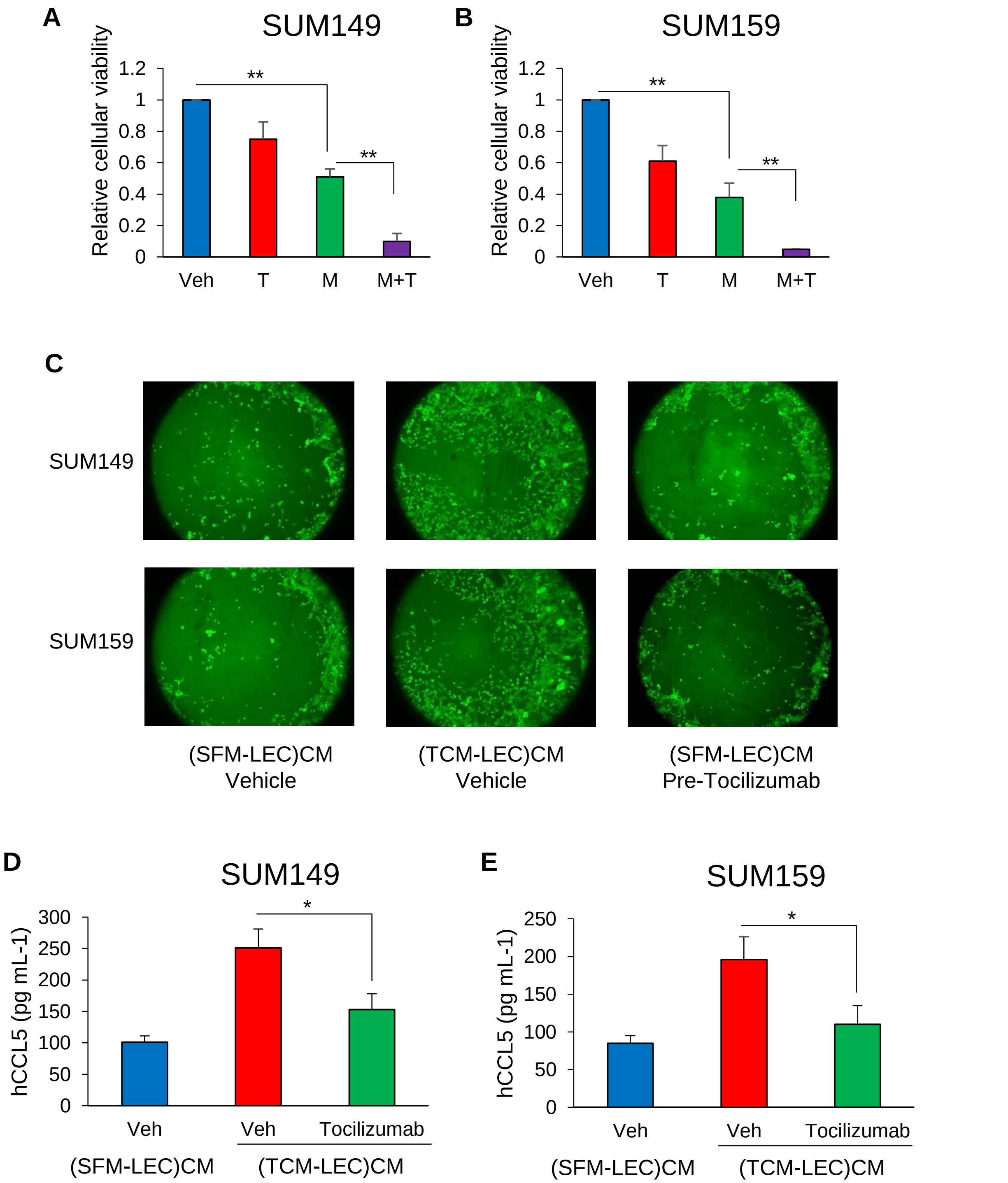

Figure S2: Migration assay of **a** SUM149 and **b** SUM159 cells (top chamber) in CM (bottom chamber) from LECs co-cultured with two TNBC cells with treatment of maraviroc, tocilizumab, and the combination. The migrated cells were counted for 24 h by the crystal violet staining. The representative migration was shown. (**P<0.001, n=3). **c** Migration assay of TNBC cells in CM from LECs co-cultured with TNBC cells with tocilizumab pre-treatment. The pre-labeled with Cell Tracker Green and the migration was measured using the Oris cell migration kit. ELISA of human CCL5 (Quantikine ELISA, R&D System) in the CM of LECs co-cultured with **d** SUM149 and **e** SUM159 cells pre-treated tocilizumab. (*P<0.001, n=3).
